# On the Elementary Organization of the Brain

**Published:** 1854-10

**Authors:** Rud. Wagner


### *On the Elementary Organization of the Brain.*

By Prof. Rud. Wagner.

(Nachr. von d. Gött Ges. d. Wiss.—Jan., S. 25.)

The physiology and anatomy of the nerves are already indebted to the masterly and exact investigations of the author for many important contributions, the discovery of new facts and the confirmation of many laws. The expectations excited in us by every new neurological treatise of the author have been most brilliantly fulfilled in the present work.

The author, with his well-known and reliable faculties of observation, has studied the properties and the reciprocal relations of the elementary forms of the nervous centres, and, with his usual acuteness, has deduced therefrom a number of new and astonishing facts and laws. Scarcely at any time, however, has a new law appeared in this branch of physiology that has not met with various opponents, and the greater the novelties the greater has been the opposition. A like fate may well befall the present work. The decussation of the anterior commissure will not be readily given up. Even if the author has deceived himself, or if the majority so consider it, with regard to certain points, yet are we not less convinced of the infinite importance of certain facts, which he brings forward as the results of his wide-spread investigations. He has adjusted, enlarged and established the basis of the nervous centres.

Our attention is directed to the great void in our knowledge of the functions of the individual parts of the brain,—the cause of which lies principally in our want of a physiological anatomy of the brain, but which can never be supplied by the rough method of vivisection. Four methods are pointed out as being those that until now have been applied with much toil, but little result, by many distinguished investigators. Some have studied the structure of the brain by numerous transverse sections in various directions. Some have sought to follow up, in hardened preparations, the fibres from the spinal marrow. Others have examined, with weak magnifying powers, segments taken from various parts and in various directions; others, again, with high magnifying powers, have analyzed more closely the elementary tissues. Little physiological information has been obtained from the history of evolution and of comparative encephalatomy, which at first promised so much. More may be expected from Pathological anatomy and from actual scientific phrenological studies. The author has made use, in his numerous investigations, of all the above mentioned methods, principally, however, the third and fourth. In investigating the brain structure, he has used, first of all, the human brain, then the brain of dogs, hares, doves, frogs, many fish, particularly the carp and the magnetic ray. The method of measuring and weighing, which he also tried, he rejects, with justice, as being in the highest degree, unreliable and deceptive; the best proof of which is, that Volkman and Kölliker arrived by it at directly opposite conclusions, with regard to the disputed termination of the nerves in the spinal marrow.

The brain contains the following elementary structures, viz :—first, primitive fibres; second, ganglion cells; third, nuclei; fourth, intermediate finely granular masses. The third and fourth can be left out of consideration, since they do not appear to stand in direct relation to the functions of the brain and are not functional nerve elements. The finely granular masses are found in the gray substance between the ganglion cells, where vessels penetrate only, and are wanting where none are present; their amount stands in direct ratio to the number of the vessels. They are intended, apparently, to prevent the dangerous contact of the dilating and expanding vessels, and serve, perhaps, as Henle maintains, as a mother-substance for the ganglion cells, with the contents of which they seem to be chemically identical. The nuclei also do not appear to be functional elements, perhaps only so where they lie in masses; according to the author, they are there analogous to the granules of the retina, not nuclei, but cells connected with fibres, (the ash-colored substance of the cortical portions of the convolutions of the cerebellum.)

With regard to the ganglion cells, the author has arrived, through numerous investigations, at the conviction, that *only stel-*

*late multipolar* ganglion cells occur in the brain; all the so-called a-polar, uni- and bi-polar, are only mutilated multipolar cells. In the great majority of the groups of ganglion cells of the brain he found a structure in close accordance with the lobes of the magnetic ray. The processes of the ganglion cells are, in part, original sources of primitive fibre, and in part serve to connect the ganglion cells together. The primitive fibres of the brain are of different diameters,—varying from 0.01 to 0.001 millimetre. The author distinguishes 1st, the thickest, from 1-200 to 1-100 m.; 2d, the moderately thick, from 1-400 to 1-300 m.; 3d, the moderately fine, from 1-600 to 1-500 m.; 4th, the fine, from 1-800 to 1-700 m.; 5th, the finest, from 1-1000 to 1-900 m. The thickest fibres occur nearly always as root-fibres of nerves; the finest, generally, towards the cortical substance of the cerebrum and cerebellum; the middle-sized ones scattered generally; the moderately thick generally dwindle to the finest towards their ending in the ganglion cells. All the fibres show axis cylinders. In general, the finest fibres pass only into ganglion cells. The axis cylinder, in fact, being metamorphosed into a process.*

All ganglion cells, from the lower ending of the spinal marrow, through the medulla oblongata and the ganglions of the middle brain, even as far as the corpus dentat cerebelli and the corpus striatus, have, according to the author, the same character and the type as found by him in the magnetic ray. They are of various sizes, variously colored (pigmented) but always provided with 4—6—15—20 processes, which either pass into primitive fibres or into neighboring ganglion cells. In a note he cites numerous individual observations, showing the positive presence of the connections of the ganglion cells by these processes.

Another system of multipolar ganglion cells are found, for instance, in the cortical substance of the cerebellum. The retort-formed ganglion cells are here present, which receive, into their bulbous end one or two moderately fine fibres, whilst, from the

* This latter view is doubtless correct with regard to the optical appearance in the dead brain, but, in our opinion is not the case in life, as it is impossible for us to determine upon the pre-formation of an axis cylinder, since we believe it to be, from many physiological and chemical grounds, a product of coagulation only.—*Funke.*

neck end or process, a great number of very fine wide-spreading twigs are given off, which pass into the finest fibrils. These cells do not appear to be connected with each other; probably each forms a system complete in itself.

The author concludes from these observations: "The following facts appear to me to be established, being the results of extended investigations. The brain and spinal marrow are nothing but massive groups of primitive fibres and multipolar ganglion cells. Combinations of primitive fibres occur only through the interposition of ganglion cells, consequently, all transitions from one primitive fibre to the other take place in the anatomical manner indicated. Gray substance, and its action upon the nerve fibres, is the obscure expression for the clear one—multipolar ganglion cells connected with primitive fibres. All the phenomena of innervation depend upon combinations of single ganglion cells and great aggregates of ganglion cells, acting as peculiar provinces of innervation, of various physiological importance among themselves, and with central and peripheral paths of nerves. The supposition of a transference from fibre to fibre, in the sense of the so-called paradoxical convulsion (Zückung) is not needed. From many reasons, that need not be entered into here, it is improbable in the highest degree; it could only act as a disturbance in the path of the physiological phenomena."

It is to be regretted that the author has not given us a special exposition of these important anatomical facts regarding the individual provinces of the nervous centres; only a few short explanations here follow, concerning some provinces of innervation particularly studied.

The fibres entering through the posterior roots of the spinal marrow unite into three principal bundles. One portion of the sensitive fibre ascends, without uniting itself with ganglion cells, to the brain, producing conscious sensation. Another part unites itself with the multipolar ganglion cells of the posterior grey horns, from whence, in part, fibres ascend to the brain, and in part, pure commissure fibres cross over behind the central canal to the ganglion cells of the posterior cord of the other side. A third portion causes no sensation, but goes to the great ganglion cells in each hemisphere by the corresponding anterior cords, from which fibres pass off to the motor anterior roots.

The author gives the following anatomical foundation for these facts; 1st, direct observation; 2d, the fact that divisions of the primitive fibres are never found in the spinal marrow, but which would necessarily be present if the same peripherical fibre ascended to the brain and at the same time went to the ganglion cell of the anterior cord; 3d, the fact that through the posterior roots more fibres are conducted into the spinal marrow than through the anterior. According to the author *none* of the fibres of the anterior roots go direct to the brain. *All* unite themselves with the great ganglion cells of the anterior horn, from which, part of the fibres pass to the brain, part pass diagonally to the ganglion cells of the other side. The *anterior commissure is therefore no decussation of the anterior cords.*

The necessary consequence of this statement is the acceptation of a system of reflex motor fibres and cells, analogous to the system of Marshall Hall, and the necessity resulting therefrom that there should be, at every point of the sensitive periphery, a sensitive and an exciting fibre. Unfortunately the author does not go further into the proof of this consequence, the necessity of which, to us, is not so apparent as he asserts. We do not venture, however, to criticise the above views, at least not until we know the author's grounds for the same, and for the refutation of the numerous arguments to the contrary. The author assumes, therefore, to proceed in his own words, that there is, in the periphery of the body, a system of simple (reinen) fibres, with geometrical unfolded terminal points, which ascend through the spinal marrow, partly direct, partly through the multipolar ganglion cells of the posterior cord (most evident, for instance, in the gustatory corpuscles of the tongue). The majority of such corresponds with geometrically arranged terminal points in the brain. Inserted between the same, lie in the periphery the likewise geometrically arranged termini of other centrifugal fibres, which, at the same time enter through the posterior roots and have their end points in a corresponding system of multipolar ganglion cells, out of which the efferent motor-fibres, in general lying at the same elevation, proceed, through the anterior roots of the corresponding half of the spinal marrow. In a note, the author totally rejects Pflüger's new doctrine of the sensorial functions of the spinal marrow—principally because repeated experiments on frogs have led him to other results.*

The author proceeds to show that the arrangements of the structures as shown above in the spinal marrow, occurs again in the medulla oblongata, only more ingenious arrangements are met with, owing to the little space. The arrangements in the brain for the central organs of the senses is more complicated, but quite analogous in its fundamental relations. In the retina the termini of the optic nerve are found explicated like the termini of the sensitive nerve in the gustatory corpuscles, though in a more delicate geometrical manner. Through the tractus opticus pass the central leading fibres of the optic nerve into the corpus geniculatum, in order to unite for the greater part with the ganglion cells. The tubercula quadrigemini are the second series of aggregates of ganglion cells with which the optic nerve unites. From these the fibres pass inwardly, enter into combination with the medulla oblongata, through the lemniscus, unite themselves with the ganglion cell apparatus on the floor of the aquaductus sylvii, and with the nuclei of the oculo-motor nerve, whose ganglion cells give off fibres not only for the corresponding muscles of the eye, but also for the circular muscles of the iris. Finally, the optic nerve

* The reviewer is among those that received Pflüger's paper with enthusiasm, and has not been cured thereof by Lotze's acuteness, nor even by the present work of the author. I have also repeated the experiments on frogs, and am engaged therewith at present; but what I have observed teaches me daily more and more that the reflex action of the phenomena upon a dead mechanism is impossible. Lotze has also perceived this difficulty, and saves himself from the necessary supposal of a mind in the spinal marrow, by an ingenious though untenable hypothesis (interesting in psychology but worthless to physiologists,) of a prolonged action of the mechanism of the spinal marrow, exerted by the prior-existing mind: an *after-action* of the intelligence. Lotze starts from the proposition, that the mind is indivisible; that is his axiom, upon which he erects his logical structure. Such a proposition may pass in psychology, but, in physiology, a-priori axioms will not do.

The author points out the identity of the principle of structure in the brain and spinal marrow. This identity can be better used in the support of Pflüger's than Marshall Hall's views. It all depends, ultimately, upon what shall be understood by the term *mind.* An idea that is indeed only too extensive, but which is covered by a veil that the physiologist dares strive to lift in a physiological manner only.—*Funke.*

fibres unite also with the cells of the thalamus, whilst from these, fibres pass into combination with the hemispheres of the cerebrum. The impression taken up by the ends of the retina fibres is therefore delivered to the ganglion cells in the corpus geniculatum, tubercula quadragemini and optic thalamus for preparation, before it is communicated to the cerebrum for the last phase of innervation, in order to enter the circle of mental perceptions as a complete ocular representation.

Thee minute anatomy of the cerebrum and cerebellum teaches us that at least the larger part of the fibres shooting through the crura of the cerebrum and cerebellum, attenuated in certain parts, end in ganglion cells, and particularly in the cerebrum, in the corpus striatum, in the last division of the nucleus lentiformis, and in the corpus rhomboideum; from this apparatus of ganglion cells arise another system of fibres, which seem to produce the reciprocal action of these central organs with the multipolar cells of the cortical substance. The author finally sums up the fundamental relations in the following proposition.

"All the phenomena of innervation in the brain and spinal marrow depend upon one of many proportions of geometrically arranged anatomical combinations of multipolar ganglion cells with one another, and upon the origin of nervous fibres from such cells, denying all direct combination of two or more fibres among themselves. Fine granular substance and nuclei play only subordinate parts.

"The problem for future physiologists is to trace back with the help of new methods, the physiological phenomena through this structure, simple in its elements, but complicated from their infinite numbers and combinations."

It is unnecessary for us to direct attention to the great value of this proposition, the admirable powers of observation of its author being a pledge for its correctness. We do not deceive ourselves in stating that a new era has begun through this work, similar to that created by Du Bois-Reymond for the physics of the nerves.—*Schmidt's Jahrbüch*, *May*, 1854.—H. P. FUNKE.

